# Eye-Head Coordination for Visual Cognitive Processing

**DOI:** 10.1371/journal.pone.0121035

**Published:** 2015-03-23

**Authors:** Yu Fang, Ryoichi Nakashima, Kazumichi Matsumiya, Ichiro Kuriki, Satoshi Shioiri

**Affiliations:** 1 Graduate School of Information Sciences, Tohoku University, Sendai, Japan; 2 Research Institute of Electrical Communication, Tohoku University, Sendai, Japan; 3 Core Research for Evolutional Science & Technology, Japan Science and Technology Agency, Tokyo, Japan; UMR8194, FRANCE

## Abstract

We investigated coordinated movements between the eyes and head (“eye-head coordination”) in relation to vision for action. Several studies have measured eye and head movements during a single gaze shift, focusing on the mechanisms of motor control during eye-head coordination. However, in everyday life, gaze shifts occur sequentially and are accompanied by movements of the head and body. Under such conditions, visual cognitive processing influences eye movements and might also influence eye-head coordination because sequential gaze shifts include cycles of visual processing (fixation) and data acquisition (gaze shifts). In the present study, we examined how the eyes and head move in coordination during visual search in a large visual field. Subjects moved their eyes, head, and body without restriction inside a 360° visual display system. We found patterns of eye-head coordination that differed those observed in single gaze-shift studies. First, we frequently observed multiple saccades during one continuous head movement, and the contribution of head movement to gaze shifts increased as the number of saccades increased. This relationship between head movements and sequential gaze shifts suggests eye-head coordination over several saccade-fixation sequences; this could be related to cognitive processing because saccade-fixation cycles are the result of visual cognitive processing. Second, distribution bias of eye position during gaze fixation was highly correlated with head orientation. The distribution peak of eye position was biased in the same direction as head orientation. This influence of head orientation suggests that eye-head coordination is involved in gaze fixation, when the visual system processes retinal information. This further supports the role of eye-head coordination in visual cognitive processing.

## Introduction

The relationship between action and visual cognition is a central issue in cognitive science and neuroscience [1, 2]. Interactions between body representation and movement and vision have been studied in a variety of experiments [3–10]. Among them, the close relationship between gaze shifts and cognition is well-known [11–14]. A typical example is Yarbus’s study, which demonstrated that tasks influence eye movements when subjects view a single picture [11]. Scan path patterns for the same picture varied dramatically for different questions. The eyes and head move in coordination during gaze shifts, and a relationship between eye-head coordination and visual cognition is expected. Indeed, such a relationship has been suggested for visual search. Nakashima and Shioiri reported that performance was better when the visual stimulus was viewed straight ahead versus viewed laterally. This illustrates the importance of eye-head coordination during visual cognition, suggesting that eye and head orientation is coordinated to shift gaze for high cognitive performance [15].

Previous studies on eye-head coordination have focused mainly on the mechanistic aspects of the motor control system using simple stimuli and tasks in laboratory environments. These studies revealed that head movement amplitude is proportional to gaze shift amplitude [16–22]. More precisely, head movement amplitude is proportional to the angle of the target with respect to the head, which is equivalent to gaze shift amplitude when the eyes and head are aligned at the initial gaze location. Stahl showed that the head moves little for gaze shifts to a target near fixation, but moves with the eyes for gaze shifts to a target far from fixation. There appears to be a certain range of gaze-shift sizes within which the head moves little or not at all (“eye-only range,” EOR). EOR varies among individuals, but is about ±18° on average [22]. It has been suggested that EOR is determined by a variety of physiological factors, such as the mechanical rotational limits of ocular motility (about ±55°) [23], and decreases in fixation accuracy and stability at far-eccentric eye positions. Most previous studies have investigated eye-head coordination during single gaze shifts, such as rapid gaze shifts from one LED light to another, which requires only limited cognitive processing [16, 18, 22–27]. Although several studies used more natural tasks, such as making tea, driving, or walking, analyses of eye-head coordination in these studies were restricted to single gaze shifts [28–35].

The purpose of the present study is to investigate eye-head coordination related to cognitive processing. When gaze shifts to look at a visual stimulus presented peripherally, head movements can be predicted by physiological factors. However, gaze often shifts sequentially in everyday life. There is no doubt that gaze shifts are influenced by information processed at the previous fixation, and control of gaze-shift sequences is likely under the influence of visual cognitive processing. Kowler et al. reported that the eyes sometimes moved in the opposite direction of head movements during reading, although the eyes and head more frequently moved in the same direction [33]. The opposite pattern of eye movements occur when a reader tries to read a word that was not identified during the previous gaze fixation. Cognitive processing should, at least occasionally, influence whether eyes move in the same or opposite direction as head movement. Investigating sequential gaze shifts is important for understanding the relationship between eye-head coordination and cognitive processing because control of gaze-shift sequences is likely under the influence of visual cognitive processing. The present study investigated eye-head coordination with unconstrained body movement during a visual search task, which was chosen as a typical task that is encountered in everyday life [36–38]. We measured eye, head, and body direction while subjects searched for a target in a wide field of view (360° display) realized by six displays surrounding them. Subjects were instructed to find a target presented in one of six displays as rapidly as possible, such that they had to actively move their body, head, and eyes except when, the target was presented in the display that subjects were initially facing.

To examine eye and head movements during visual search, we conducted two analyses that were very different from those used for single gaze shifts. First, we analyzed the number of saccades in a single head movement. There may be multiple saccades during one head movement when gaze shifts sequentially, such as during reading. The head tends to keep moving from the left to right while reading a sentence (in English) [33–35]. Some studies have revealed that cats make multiple saccades during one head movement [39, 40]. There are also reports of secondary gaze shifts during single head movements in studies that used natural tasks in humans, such as reading, making tea, driving, or walking, while the secondary gaze shifts may be regarded as corrective saccades [21, 28, 33–35]. Multiple saccades during one continuous head movement may not be rare, but this has not been investigated extensively. Second, we obtained the eye position distribution for different head orientations. Since no fixed relationship between the eyes and head is expected, the relationship between eye position and head orientation can be best described probabilistically as the effect of head orientation on the distribution of eye positions. Theoretically speaking, eyes can be directed at any point within the anatomical limit of the oculomotor system wherever the head is oriented. EOR, observed in studies with simple stimuli and tasks, is evidence for the large distribution of possible eye directions with respect to head direction [22]. However, Nakashima and Shioiri suggested that visual search performance is best when the eyes are oriented in the same direction as the head [15]. This could indicate a systematic relationship between head orientation and eye position distribution under free viewing conditions. We analyzed saccade number during one head movement and the eye position distribution for a given head orientation to explore the relationship between eye-head coordination and cognitive processing. Note that we did not analyze systematic gaze characteristics such as distribution of gaze location, gaze duration, and scan path because our primary interest is eye-head coordination. These analyses are beyond the scope of the present study.

## Materials and Methods

### Subjects

Eight human subjects (1 female, 7 male, aged 22–31 years) with normal or corrected to normal vision (only contact lenses were allowed to avoid interference with the eye tracker) performed the experiment. There was only one female subject, suggesting a possible gender bias, but to our knowledge there are no reports of gender biases on eye-head coordination. Moreover, there were no systematic differences between the female and male subjects’ data, and related studies on eye-head coordination showed similar results for male and female subjects [27, 41]. All subjects were students or staff of Tohoku University and all except one (S2, who is one of the authors) were naïve to the purpose of the experiment. The experiment was approved by Tohoku University’s institutional review board (H24–2), and all subjects gave written informed consent before participation.

### Experimental setup

**Fig 1:**
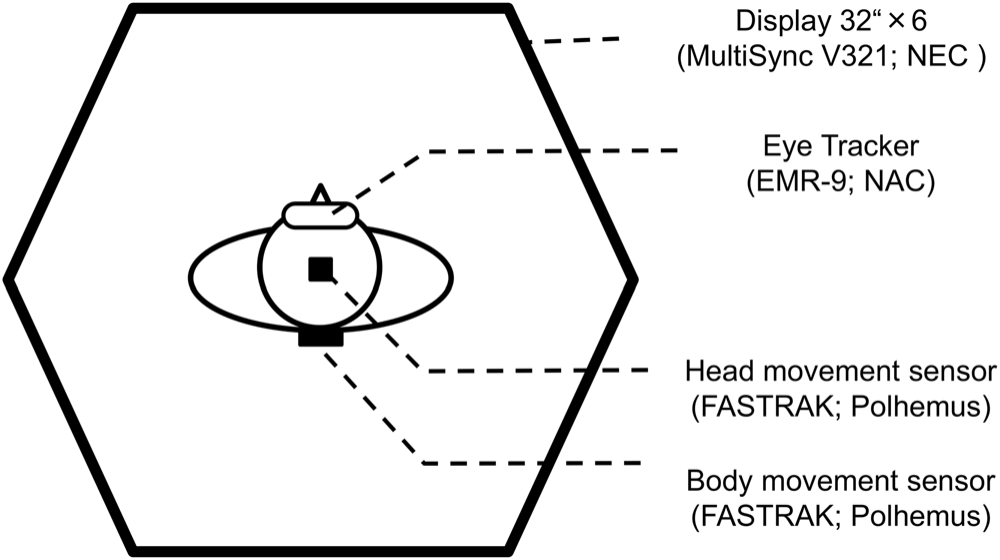
Experimental setup. Experiments were performed in a dark room using six 32-inch liquid crystal displays (LCDs; MultiSync V321; NEC, Japan; 60 Hz), arranged in a hexagon. Eye movements were recorded by an eye tracker (EMR-9; NAC, Japan; 60Hz). Head and body movements were recorded by two small, light-weight sensors from an electromagnetic motion tracking system (FASTRAK; Polhemus RS-232, USA; 60 Hz).

The experiment was performed in a dark room using six liquid crystal displays (LCDs; MultiSync V321; NEC, Japan) with a refresh rate of 60 Hz, arranged in a hexagon (Fig. 1). The displays were centered at a height of 155 cm, which was slightly higher than the average height of subjects’ eyes from the floor (150 cm). An electromagnetic motion tracking system (FASTRAK; Polhemus, USA) was used to measure the orientation (azimuth, elevation) of two small, light-weighted sensors. One sensor was placed on the subject’s head, and the other was fixed on the subject’s back to record the orientation of the head and body in space at a 60 Hz sampling frequency with 4 ms latency and 0.15° of accuracy. Eye-in-head positions were recorded at 60 Hz by an eye tracker (EMR-9; NAC, Japan), which contains three cameras; two recorded the positions of the two eyes, and the scene camera in the middle had a field-of-view of 62° visual angle. The eye tracker has 71 ms delay and 0.1° of accuracy in measurements. All eye, head, and body orientation signals were synchronously recorded by a computer, which also controlled the stimulus displays. The display was arranged 60 cm from the center of the area where the subject moved around during trials.

### Stimuli

**Fig 2:**
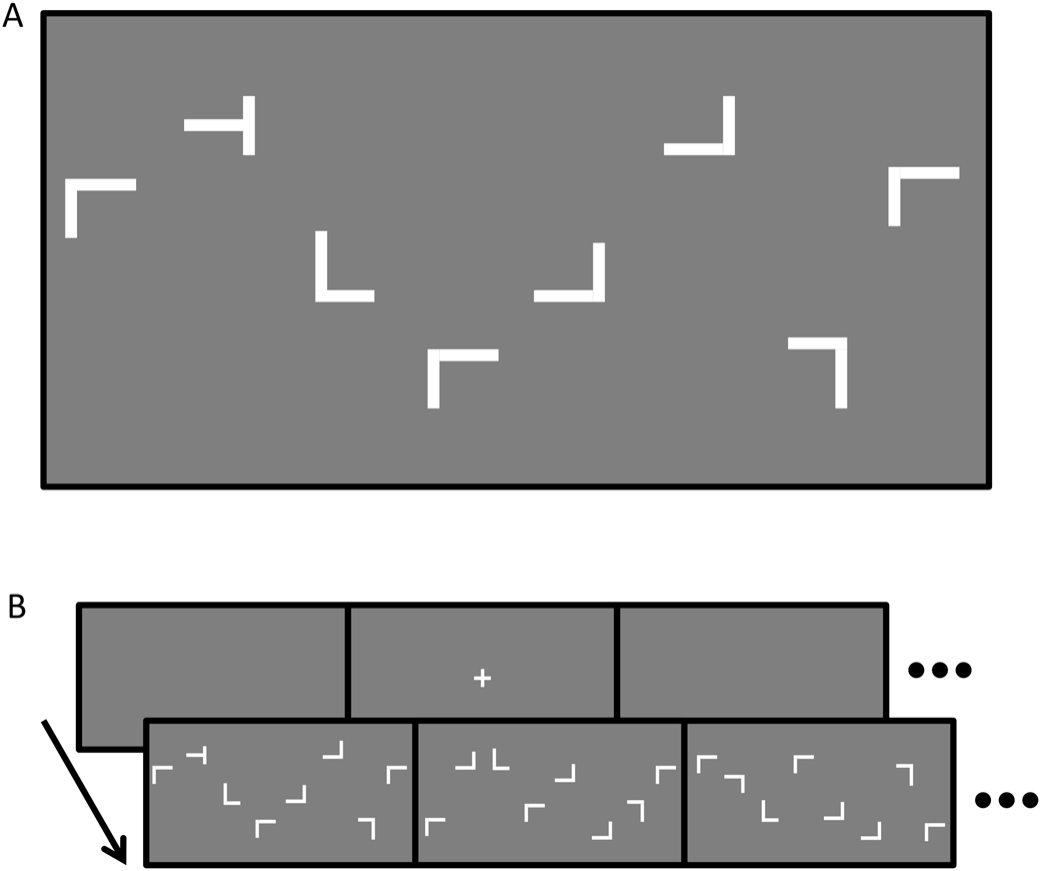
Example of visual search stimuli. (A) White target and distractors were presented randomly on a gray background in one display; (B) A typical frame presentation sequence for one trial.

The task was to search for a target (“T”) among 47 distractors (“L”). The target and distractors were distributed among the six displays in random arrangements with the restriction that eight items were presented in each display (about 60° × 34°). The target “T” was rotated 90° to either the left or right, while distractor “Ls” were rotated by 0°, 90°, 180°, or 270°. The target and distractors were white (325.6 cd/m^2^) and were presented on a gray background (63.8 cd/m^2^). The target and distractors were 1.3° × 1.3° (Fig. 2A). Stimuli were generated using the Psychophysics Toolbox [42, 43] for MATLAB (Mathworks, U.S.A.).

### Procedure

Fig. 2B illustrates the stimulus presentation for one trial. A fixation cross was presented at the center of the front display at the beginning of each trial, and the remaining displays were blank. One display was used as the front display throughout the experiment. Standing at the center of the displays, the subject faced this front display to look at the fixation cross; then he/she pressed a button to start the trial. After a 500 ms delay, the fixation cross and blank screens were replaced by the stimulus layout. Subjects were instructed to search for the target that was located in one of the six displays as rapidly as possible. They moved their head and body freely during search. When they found the target, they reported its orientation (left or right) by button-press. Ten practice trials preceded the experimental trials. There were four blocks of 48 trials. Subjects took a break for about 5 minutes after the first two blocks.

The eye tracker was calibrated before the experiment and the position tracking system was calibrated before each block. Eye and head movement data were recorded during the experiments and analyzed later.

### Data analysis

Eye positions were recorded as the angle of eye-in-head. For head and body movement, the head position relative to the body was obtained by the difference between head-in-space and body-in-space (both azimuth and elevation angles). Gaze position—where the subject was actually looking—was derived by the sum of the eye-in-head and head-in-space values.

**Fig 3:**
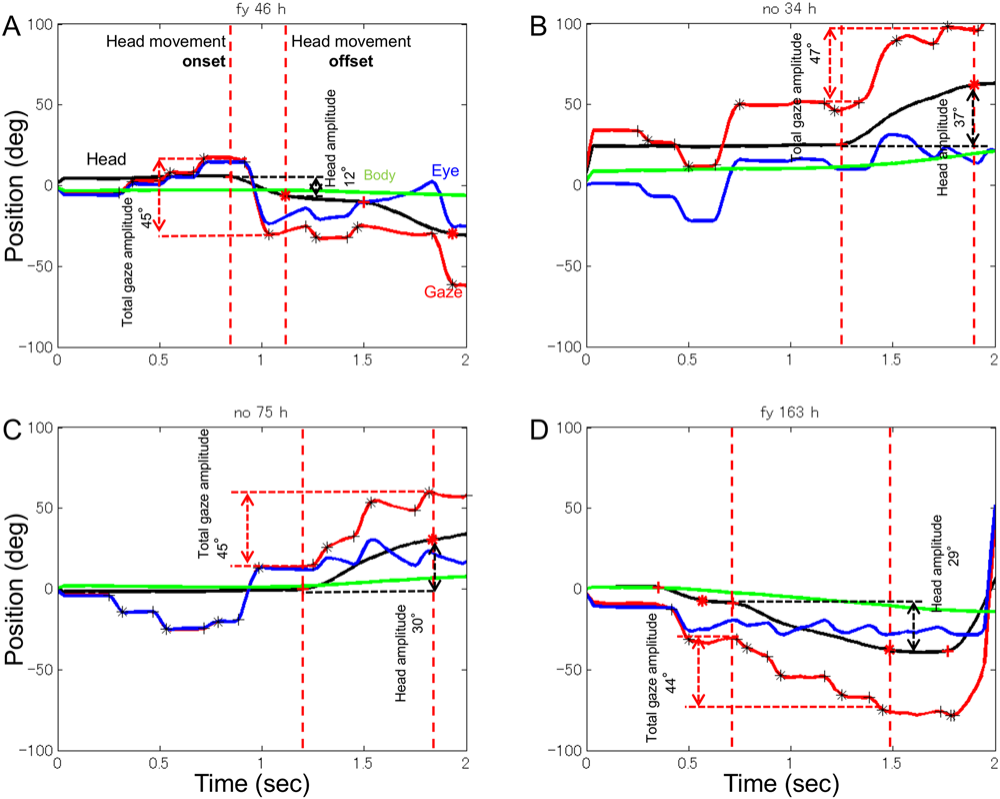
Example horizontal eye and head movement trajectories. Four examples of eye (blue), head (black), body (green), and gaze (red) rotational trajectories during the first two seconds of each trial. The plus and star indicate the onset and offset, respectively, of each gaze and head movement. A saccade is the distance between the plus and star. Upward deflection of the traces indicates rightward movement. The two vertical dashed lines across each trace represent head movement onset and offset. One saccade (A), two saccades (B), three saccades (C), and four saccades (D) in one head movement are shown.

To determine the onset and offset of eye and head movements, the speed and acceleration of gaze and head movements in two dimensions were calculated. Saccade onset was defined as the time at which both gaze speed and acceleration exceeded the threshold values of 75°/s for speed and 200°/s^2^ for acceleration. Saccade offset was defined as the time at which both speed and acceleration fell below threshold values. Similarly, head movement onset and offset were defined using speed and acceleration thresholds of 20°/s and 50°/s^2^, respectively (Fig. 3A). Horizontal and vertical gaze shift and head movement amplitudes were defined as the horizontal and vertical angular difference between onset and offset locations. Fixation position was calculated by averaging the eye-in-head data over the fixation period (between the offset of a saccade and the onset of the next saccade), and head orientation was calculated by averaging the head-on-body data over the same gaze fixation period. Data from one subject were discarded because of errors in eye movement recording likely due to calibration problems.

Multiple saccades include cycles of fixation and gaze shifts during a single head movement. To investigate the effect of saccade number on eye-head coordination, we first determined gaze shift and head movement onsets and offsets. Next, for each head movement, we considered a saccade to be related to the head movement when either the onset or offset occurred during the head movement. The gaze shift amplitude during the head movement was defined as the total gaze shift of all saccades during the head movement. Saccades during stationary head were also analyzed separately.

## Results

Fig. 3 illustrates four example horizontal eye and head movement trajectories during the initial 2 seconds of four different trials. At the beginning of each trial, subjects scanned items on the front display with few head movements, and gaze location was virtually identical to the eye-in-head location. After searching the initial display, the head usually moved in coordination with the eyes to search other displays. On some occasions, there was a single movement of the head and eyes, where the head and eyes moved in coordination as reported in previous studies of single gaze shifts (see Fig. 3A). On other occasions, however, there were multiple saccades during one head movement (see Fig. 3B, 3C, and 3D). This type of coordinated eye and head movement is very different from eye-head coordination during single gaze shifts.

**Fig 4:**
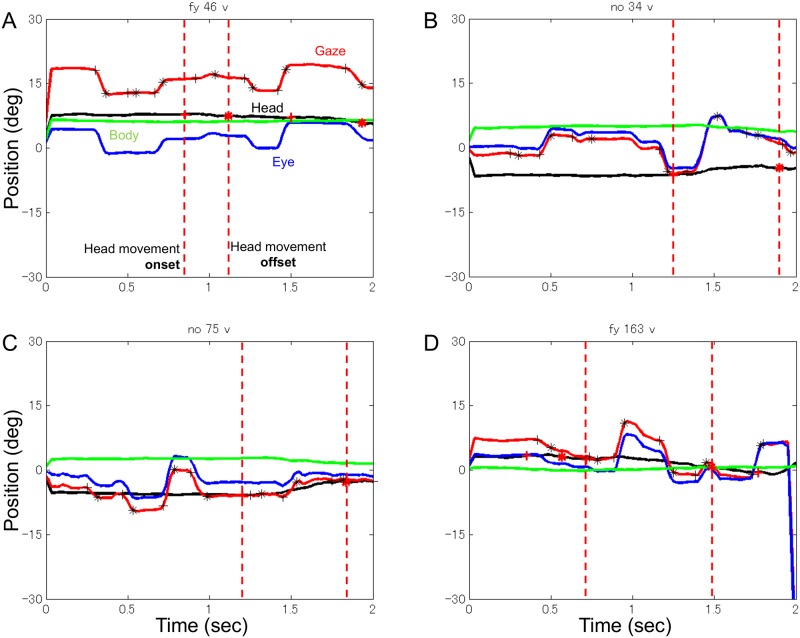
Vertical eye and head movement trajectories. Four examples from the same trials as Fig. 3. Notations are the same as in Fig. 3, but upward deflection of the traces indicates upward movement.

Fig. 4 illustrates four example vertical eye and head movement trajectories for the same four different trials. Because the onset and offset of gaze and head movements were determined in two dimensions, both horizontal and vertical components share the same onset and offset for each gaze and head movement. Compared to the horizontal component, in the vertical component the eyes and head moved little, and the eyes moved more flexibly relative to the head (see Fig. 4D).

**Fig 5:**
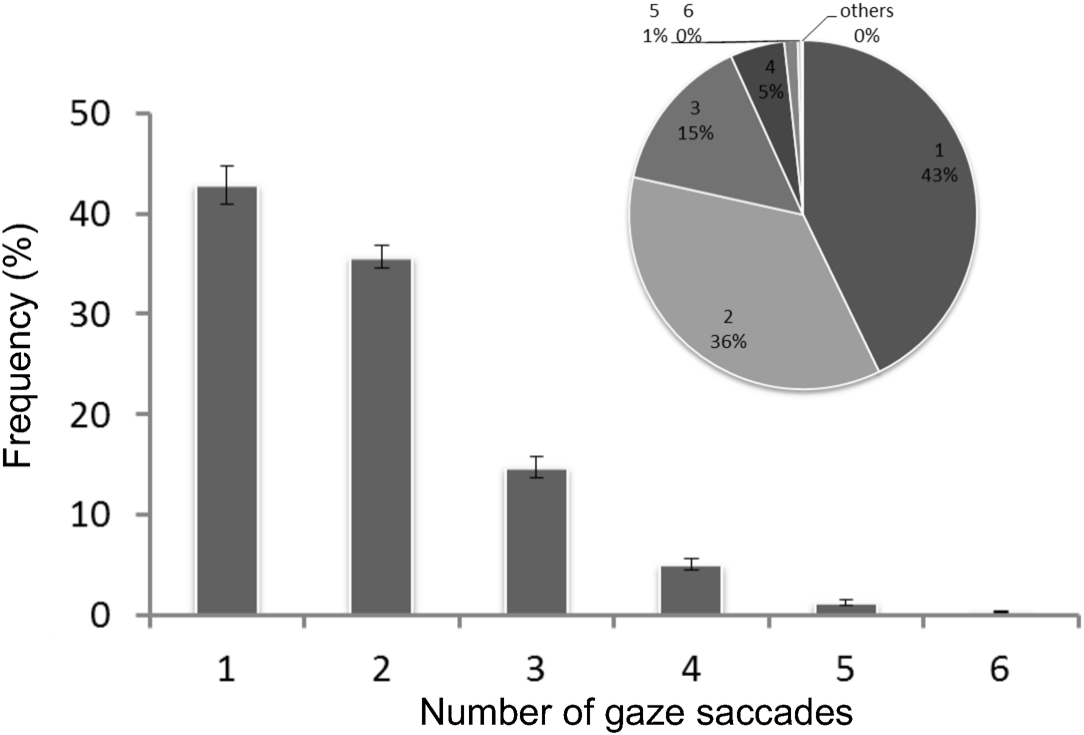
Percentage of head movements with different numbers of saccades. The bars indicate the average and standard error for seven subjects. The same data are also plotted in a pie chart to show that a larger percentage of head movements had multiple versus single saccades.

The number of saccades during one head movement ranged from one to more than four. Fig. 5 shows the percentage of head movements for different numbers of saccades. About 43% of head movements had one saccade; head movements with multiple saccades occurred more frequently (see pie chart in Fig. 4). Head movements with two saccades occurred less frequently (about 36%) than those with a single saccade, and the frequency of head movements decreased as the number of saccades increased. To our knowledge, this is the first quantitative report of eye-head coordination with multiple saccades during one head movement.

### Contribution of head movement to gaze shifts

Typical vestibulo-ocular reflexes, VORs, are observed when gazing at a visual stimulus during head movement (Fig. 3): eye movement compensates for head rotation to maintain fixation. Occasionally, however, we found drift-like movements during fixation, which appear to be under- or over- compensation for head movements by VOR (in Fig. 3C, gaze at about 1.4 s and 1.7 s show over-compensation and under-compensation, respectively). These drift-like VORs can be attributed to body movements relative to the display. If the participant moved back and forth with respect to the display, the change in distance from the fixated target changes the angle to the eye without head rotation. VOR is required to keep fixating a location with lateral viewing (i.e., eye direction differs from head direction). We confirmed that the apparent drift during fixation did not influence saccade extraction by changing the criterion for gaze shifts. The lower the threshold speed, the more apparent drift was included in a saccade (instead of VOR during a fixation), but the variance is less than 2% of total saccades when criterion speed varied from 50°/s to 100°/s and this is not expected to influence the general trends in the results. All the results shown were analyzed with a threshold speed of 75°/s, in which saccades included few drift-like movements, and actual saccades were not incorrectly treated as fixations (see Fig. 3).

**Fig 6:**
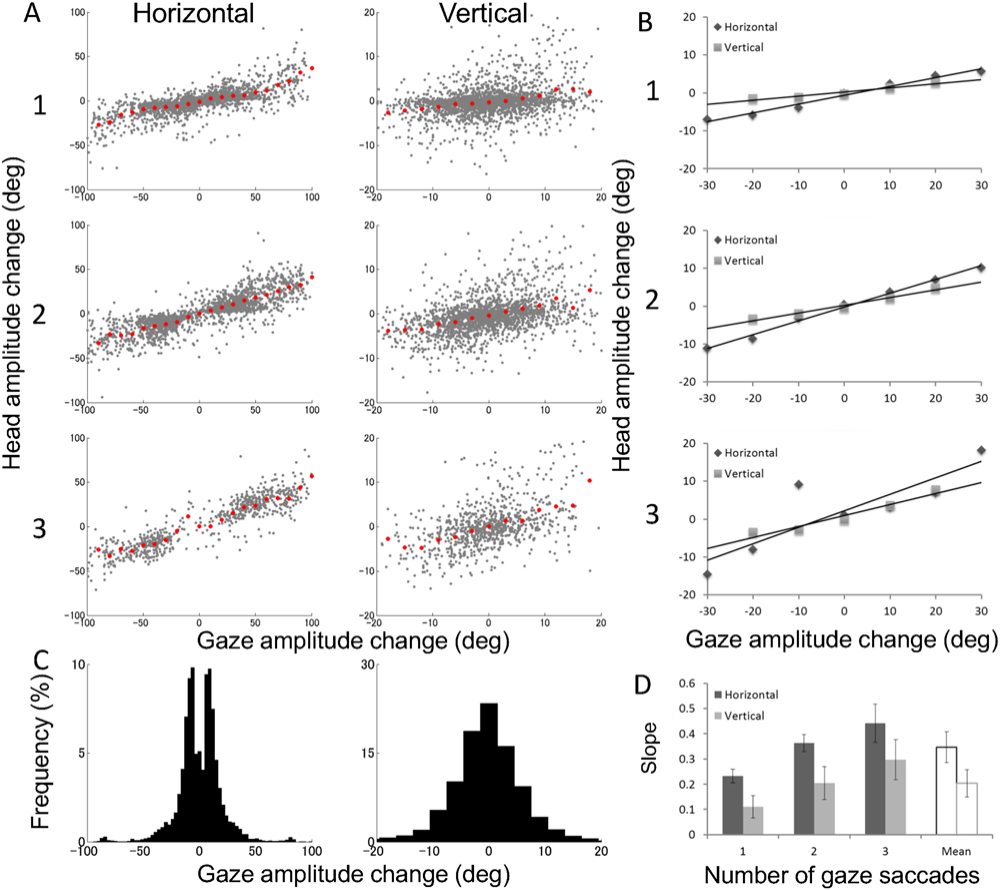
Contribution of head movement rate to gaze shifts. (A) Head movement amplitude against the total gaze shift amplitude for different numbers of saccades in horizontal and vertical components for seven subjects. Each gray dot shows the individual head movement with saccades. The red dots indicate the average data, which are binned in 10° for horizontal and 3° for vertical. (B) Comparison between average horizontal and vertical movements. Lines are linear functions fitted to the average data across subjects. (C) Distribution of horizontal and vertical gaze amplitudes without head movements for all seven subjects. (D) Average slopes for head movements with different numbers of saccades for each subject. Error bars represent standard error.

We analyzed the contribution of head movements to gaze shifts based on previous studies [18, 19, 22] that reported EORs of about ±18° and a constant contribution of head movement to gaze beyond the EORs. Fig. 6A compares head movement amplitude against total gaze shift amplitude for different numbers of saccades for horizontal and vertical components. Total gaze shift amplitude was defined as the difference between gaze onset and offset positions during a single head movement (Fig. 3). In Fig. 6A, rows indicate head movements with different numbers of saccades. The results were similar for all seven subjects and pooled data are shown. Three regions with different slopes were observed for head movements with one saccade (see the average head amplitude indicated by red dots, center range within ±50°), a pattern similar to that found in single gaze-shift experiments. The distribution of gaze amplitudes without head movements was also analyzed and is shown in Fig. 6C. Most of gaze shifts without head movements (90%) were within amplitudes of ±30° horizontally or ±12° vertically for each direction of all subjects. This is consistent with ranges of EORs previously reported. Fig. 6C shows that the shape of the gaze distribution differs between the horizontal and vertical components: double peaks are seen in horizontal distribution whereas a single peak is seen at the center in vertical distribution. This is because there were more numbers of horizontal than vertical gaze shifts in general, and there were more gaze shifts without a vertical component than without a horizontal component.

Importantly, the slope in the central region changed with the number of saccades: the more saccades, the steeper the slope. Slope magnitude indicates the degree to which head movement contributed to gaze shift: a slope of one indicates 100% and a slope of zero indicates 0%. For quantitative analysis, we obtained the slopes by fitting a line to average head movement amplitude for each gaze shift amplitude bin (3° separations; red dots in Fig. 6A and 6B). For slope estimation, we chose gaze shift ranges of ±30° and ±20° as the central region for horizontal and vertical components, respectively, based on the histogram of gaze amplitudes without head movement (Fig. 6C). The slope of the central region was estimated for individual subjects and the average across subjects with standard errors are plotted in Fig. 6D. Slope magnitude varied systematically across different numbers of saccade for both directions. Slope magnitude was 0.23 in the horizontal component and 0.11 in vertical component for one saccade, and 0.44 and 0.30 for three saccades. An ANOVA showed significant differences in slope between head movements with different numbers of saccades in both the horizontal [*F*(2,12) = 5.83; *p* < 0.05], and vertical [*F*(2,12) = 4.03; *p* < 0.05] components. We also found that the contribution of head movement to gaze shifts was larger in the horizontal than vertical component [*t*(6) = 3.25, *p* < 0.05].

We further investigated two features of eye-head coordination. First, we analyzed the similarity of movement direction between the head and gaze. Inspection of head and gaze trajectories suggests that the direction of sequential saccades and head movements are often the same when multiple saccades occur during one head movement (Fig. 3B–3D). For example, if gaze shifts in a particular direction to search for the target, sequential saccades and head movements in the same direction would be efficient for search. This was confirmed by analyzing the similarity between saccade and head movement directions. The majority of sequential saccades were in directions similar to the head movement. The percentage of head movements with multiple saccades, whose direction ranged from ±45° with respect to the direction of head movement, was 88.3%. This is approximately the same as for head movement with a single saccade (89.7%). We also compared horizontal and vertical gaze and head movement directions. In the horizontal component, 91.77% of gaze shifts were in the same direction as head movement, and in the vertical component 63.81% of gaze shifts were in the same direction as head movement [*t*(6) = 12.7, *p* < 0.001].

**Fig 7:**
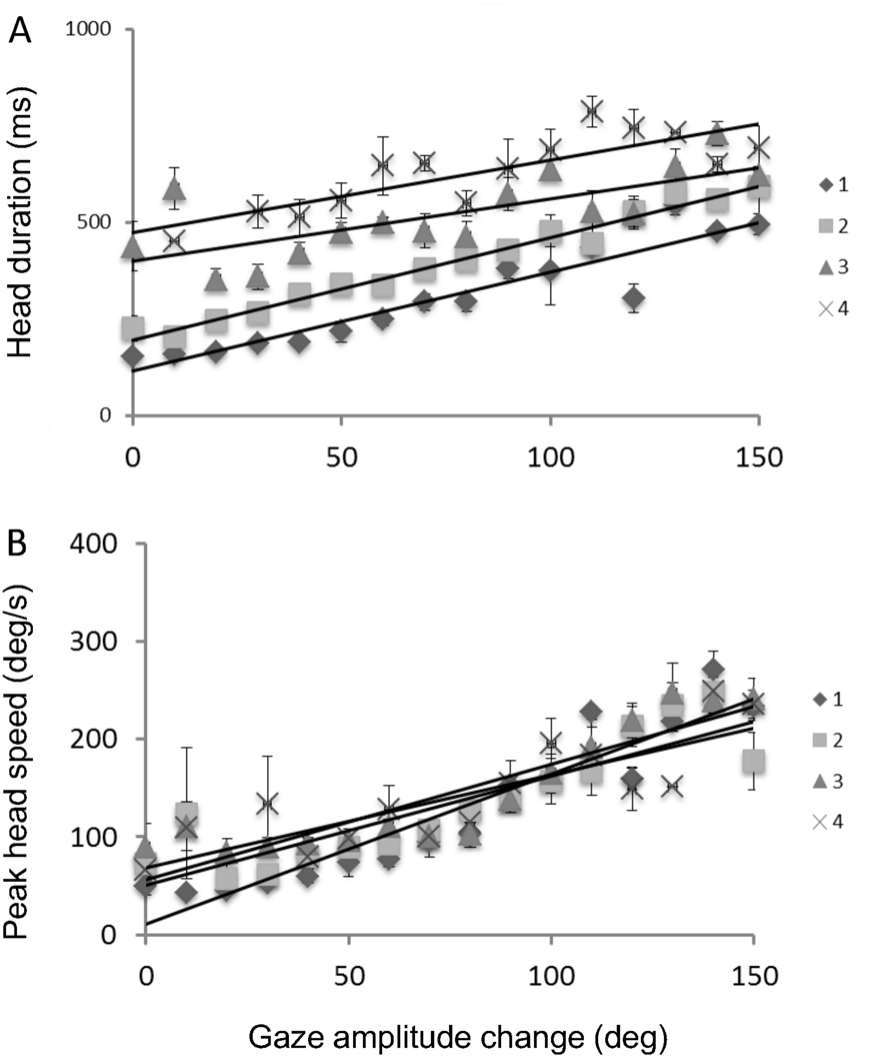
Average duration and speed of head movements as a function of gaze shifts. (A) Head duration, and (B) maximum speed plotted against total gaze amplitude change. Different symbols represent head movements with different numbers of saccades. Lines are linear functions fitted to the average data across subjects.

Second, we analyzed head movement speed and duration. Fig. 7 shows (A) duration and (B) maximum speed as a function of gaze shift size. Different symbols represent different numbers of saccades. An effect of gaze shift was found for both duration and maximum speed, which both increased as gaze-shift increased for all saccade numbers. This increase in peak head movement speed with increases in gaze amplitude is consistent with previous studies [24, 28]. There was also an effect of saccade number, but only on duration. Peak head movement speed is approximately constant for a fixed gaze shift magnitude, and is barely influenced by the number of saccades.

The proportional relationship between head movement duration and gaze amplitude is not consistent with previous studies. Previous experiments in which subjects were asked to shift gaze between two targets presented at separate locations showed that head movement duration was around 400 ms, independent of gaze shift size [24, 28]. In the present study, head movement duration varied between 200–800 ms, depending on gaze amplitude and the number of saccades. This difference may be driven by multiple saccades. Multiple saccades within a single head movement may increase head movement duration. One fixation-saccade cycle takes at least about 200 ms, and more saccades require more time.

### Influence of head orientation on eye position distribution

**Fig 8:**
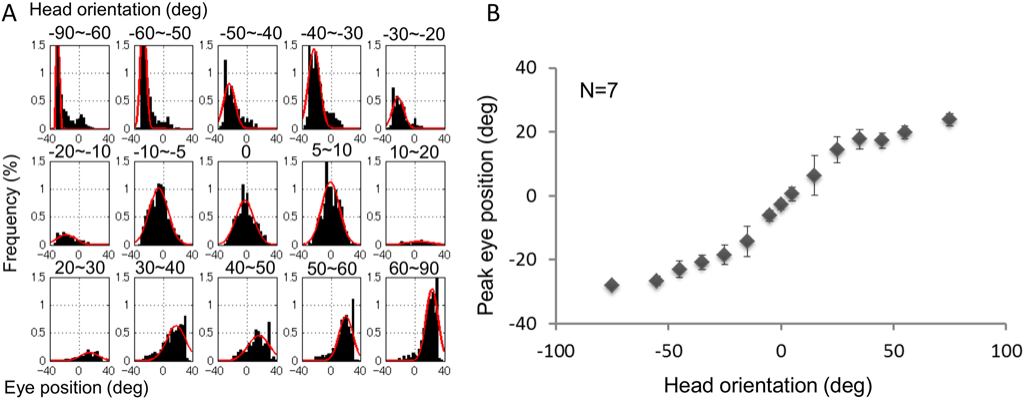
Influence of head orientation on eye position distribution. (A) Distribution of eye positions with different head orientations is shown in different panels. The horizontal head orientation is binned into 15 groups and eye position data are analyzed for each group. The range of head orientation for each group is shown at the top of each panel. The ordinate shows the percentage of fixations from all head directions and the abscissa shows horizontal eye position relative to the head. The red line shows a Gaussian function fitted by a least square method. (B) The average of the peaks of the Gaussian functions fitted to each data set from seven subjects as a function of head orientation. Error bars represent standard error.

Eye-head coordination reported in the literature is for gaze shifts or saccades [19, 22, 23, 25, 28, 31, 44]. We analyzed fixation distribution because we were interested in eye-head coordination during fixations, when the visual system processes retinal information. We used data when the head was stationary, which we expected to be influenced more by cognitive processes. The fixation distribution during head movements may include effects of a movement control process. Fig. 8A shows the distribution of eye positions for different head orientations in separate panels. Horizontal head orientation is binned and noted at the top of each panel. The ordinate shows the percentage of fixations, and the abscissa shows horizontal eye position relative to the head. To approximate the distribution, we fitted a Gaussian function to each set of data using a least squares method. The red line shows the function fitted to the average across all subjects in Fig. 8A, but we fitted the function separately to individual data for the statistical analysis.

The distribution of eye positions was dramatically biased by head orientation. When the head was orientated left (or right), the eyes also tended to be directed to the left (or right) relative to the head. To illustrate this effect, we plotted the peak eye position of the distribution (i.e., Gaussian function) averaged over subjects as a function of head orientation (Fig. 8B). Fig. 8B shows a clear relationship between peak eye position and head orientation. The peak monotonically shifts with head orientation, and the effect is similar on the left and right, that is, symmetric with respect to the origin. When the head was within ±15°, the effect of head orientation on eye position was large in terms of distribution peak, and had a steeper slope. A head orientation shift of 1° caused a peak shift in the eye position distribution of approximately 1°. When the head was outside the ±15° range, the effect became weaker (i.e., slope was shallower); a head orientation shift of 1° caused a peak shift in the eye position distribution of approximately 0.15°.

It should be noted that the measurable range of the eye tracker we used was limited to ±31° from the head center. Because of this limitation, peak estimation was performed with little information on either side of the peak for data where head orientation was large (> 60°). However, we believe that the accuracy of peak estimations here is sufficient for the present analysis because the estimated peaks change continuously from the peaks for smaller head orientations (30°–50°).

## Discussion

Using a visual search experiment in which the eyes, head, and body were allowed to move without restriction, the present study revealed three novel characteristics of eye-head coordination: 1) multiple saccades during a single head movement; 2) difference in eye-head coordination between the horizontal and vertical directions, which has been suggested, but not systematically analyzed [21, 28, 32–35]; and 3) an effect of head orientation on the distribution of eye positions when the visual system is processing retinal images. We discuss the relationship between these three characteristics and cognitive processing below.

### Multiple saccades during a single head movement

The present experiment showed that single head movements with multiple saccades constituted as much as 57% of the total head movements with saccades (Fig. 5). These results are very different from the findings of previous studies using simple tasks, where eye-head coordination was derived from a single head movement with one saccade [18, 19, 22, 24, 25, 27]. The eye-head coordination shown for single gaze shifts with a simple task is likely related to estimating the cost and benefit of head movements [27]. In contrast, we consider the possibility that eye-head coordination with multiple saccades is related to cognitive processes.

Fig. 6 shows that the contribution of head movement increased as the number of saccades increased in both the horizontal and vertical components. This indicates that the head moved more for gaze amplitudes of similar size when there were more saccades during the head movement. Typical examples are shown in Fig. 3. The head movements indicated by the two dashed red lines in Fig. 3A and 3D are similar in gaze shift amplitude. Fig. 3A shows a 45° gaze shift with one saccade during a 12° head movement, and Fig. 3D shows a 44° gaze shift with four saccades during a 29° head movement. Interestingly, eye position in the head tended to oscillate less with more saccades. Fig. 3A shows that the largest change in eye position was about 20°, whereas Fig. 3D shows the largest change of about 8°. We may expect better cognitive performance with more saccades if we assume that visual processing is stable when eye position is constant compared with larger oscillations of eye position. When performing a task such as visual search, the visual system may try to minimize eye movement amplitude by increasing the number of saccades to preserve cognitive performance. This is one possible explanation for multiple saccades during a single head movement, but this is purely speculative. Further studies are necessary to investigate the issue.

Multiple saccades were independent of head movement speed (Fig. 7B), while the duration of head movements increased as the number of saccades increased (Fig. 7A). This suggests that head movement is determined by considering the number of saccades. Because duration varies without changes in speed, duration changes cannot be attributed to the same type of head movement control for single gaze shifts, where gaze amplitude increases as duration and speed increase [19, 24, 27, 28]. We speculate that this eye-head coordination for multiple cycles is related to cognitive processes because target identification processes and/or visual strategy during visual search likely occur during the head movement period. This is more support for eye-head coordination for cognitive processes.

Observing eye-head coordination during visual search is consistent with previous physiological studies on cats [39, 40]. Bergeron et al. showed that cats make multiple saccades during one head movement, and the cat superior colliculus encodes overall amplitude of gaze displacement with the head movement, not the amplitude of individual saccades in the sequence [39]. This suggests that coordinated eye and head movements when there are multiple saccades are controlled as a single complex action. A cognitive process may program such a complex action involving multiple saccades to preserve cognitive performance, as suggested above.

### Comparison between horizontal and vertical gaze and head movements

Differences in eye-head coordination between the horizontal and vertical directions have been reported in the literature [16, 21, 25, 35]. On the one hand, the present results showed that the effect of saccade number was similar between horizontal and vertical head movements. On the other hand, we found two differences between the two directions. First, the contribution of head movements to gaze shift was larger in the horizontal (35%) than vertical (20%) component, consistent with Tweed and Vilis [25]. Second, the directions of eye and head movements differed more often along the vertical (46.2%) than horizontal (8.2%) axis. This is consistent with Goossens and Van Opstal [21], who reported that there was sometimes only one saccadic position change in the vertical component, whereas there were two saccadic position changes in the horizontal component, due to one oblique and one horizontal saccade during a single head movement. Both their results and ours suggest differences in control of horizontal versus vertical eye movements with the same head movement.

The present results, together with previous studies, suggest independent control of vertical and horizontal eye movements. Whether they share a common eye-head coordination process or not remains an open question. Similar effects of saccade number on head movement amplitude found in the present experiment (Fig. 6D) may support a common eye-head coordination process, while the difference in direction similarity between the eyes and head may indicate two control systems—one perceptual and one cognitive—for eye-head coordination [34]. The similar characteristics of horizontal and vertical movements may originate at a high-level cognitive process, and the different characteristics may originate at a low-level perception/sensation process.

### Eye-position distribution under different head orientations

We analyzed the distributions of eye position as a function of head orientation when the head and eyes were stationary. The distribution of eye positions was dramatically biased towards head orientation. When the head was orientated to the left (or right), the eyes also tended to be directed to the left (or right) relative to the head (Fig. 8). This bias is consistent with the effect of head orientation on visual cognition reported by Nakashima and Shioiri, who showed that visual performance was higher when the head and eyes were oriented in same versus different directions [15]. The distribution bias may be a consequence of trying not to orient the eyes in a direction that is largely different from the head to minimize misalignment of the head and eye. The distribution bias during fixations is consistent with the idea that eye-head coordination is related to cognitive processing because retinal images are processed during fixation. This differs from the eye-head coordination process during gaze shifts [28–35]. The eye position distribution results suggest that coordinated control of the eyes and head occurs not only for gaze shifts, but also for gaze fixations when the head still and when the visual system processes retinal information.

The analysis of fixation distribution is also important for predicting attentional focus. The distribution results indicate that head orientation provides some information about fixation location, which is usually indicative of where subjects are attending. This information can be used to predict the focus of attention in combination with numerical models of attention [45]; we have proposed a method for improving predictions of attention focus using the relationship between eye and head orientations [46].

## Conclusions

We investigated eye-head coordination during visual search inside a 360° visual display system. The experiment reveals, first, that there are frequently multiple saccades during a single head movement, and, second, that eye position is influenced by head orientation, such that the peak of the fixation distribution is biased toward head orientation relative to the body. Both findings indicate that eye-head coordination operates over several saccade-fixation sequences, including fixation periods when the visual system processes retinal information. We conclude that there is an eye-head coordination system for visual cognitive processing and one for motor control.

## Supporting Information

S1 Data Datasets of the eye, head, and body movements, including seven subjects participated in this experiment. The individual data was written in one CSV file. Each trial starts from the time of zero.
(RAR)
